# Internet addiction and appearance anxiety among university students in Shanghai: upward social comparison as a mediator and autonomous participation in physical activity as a moderator

**DOI:** 10.3389/fpubh.2026.1852639

**Published:** 2026-06-11

**Authors:** Xinxin Huang, Jiatian Qi, Huiping Dou, Xi Chen, Yue Xi, Yueyi Jia, Qiaoli Xiao, Jingjing Li, Hui Qiu, Bing Liu

**Affiliations:** 1 Physical Education College, Shanghai University, Shanghai, China; 2 School of Economics, Shanghai University, Shanghai, China; 3 College of Physical Education, Huangshan University, Huangshan, China; 4 Department of Physical Education, Shanghai Ocean University, Shanghai, China; 5 School of Physical Education, Shanghai University of Traditional Chinese Medicine, Shanghai, China

**Keywords:** appearance anxiety, autonomous participation in physical activity, internet addiction, university students, upward social comparison

## Abstract

**Introduction:**

Appearance anxiety is increasingly common among university students in the digital era, yet the mechanisms linking internet addiction to appearance anxiety remain unclear.

**Methods:**

Using survey data from 522 undergraduates from five universities in Shanghai, we tested a mediation model in which upward social comparison explains the association between internet addiction and appearance anxiety, and a moderated mediation model in which autonomous participation in physical activity (APPA) buffers this pathway. Analyses were conducted with Hayes’ PROCESS (Models 4 and 58), controlling for demographic variables and daily internet use time.

**Results:**

(1) Internet addiction was not directly associated with appearance anxiety. However, upward social comparison accounted for a significant indirect association between Internet addiction and appearance anxiety, with an indirect effect of 0.105, representing 56.15% of the total effect. (2) APPA moderated both the association between Internet addiction and upward social comparison and the association between upward social comparison and appearance anxiety. Specifically, these associations were weaker among students with higher levels of APPA.

**Discussion:**

(1) Internet addiction is indirectly associated with appearance anxiety through upward social comparison. (2) APPA may buffer this indirect pathway by weakening the associations between Internet addiction and upward social comparison, and between upward social comparison and appearance anxiety. These results highlight the potential value of addressing maladaptive social comparison in online contexts and promoting autonomous engagement in physical activity in prevention and intervention practices.

## 1 Introduction

Appearance anxiety has emerged as a global mental health issue and, as a salient socio-psychological phenomenon that transcends national and cultural boundaries, has attracted widespread attention (1). Evidence indicates that, in recent years, appearance anxiety has become a particularly prominent psychological problem among university students, gradually evolving into a new focal topic in campus mental health education (2). A survey conducted by the China University Media Alliance among 2,063 Chinese university students found that nearly 60% reported varying degrees of appearance anxiety, with 19.41% exhibiting severe symptoms. Notably, the problem shows trends toward younger onset and broader prevalence, thereby drawing substantial public concern (3). More importantly, appearance anxiety may exert sustained and multidimensional adverse effects on university students’ psychological and physical development. They can trigger excessive self-focused attention, negative self-evaluation, and social avoidance, which in turn undermines self-esteem and impairs interpersonal relationships (2). They may also motivate extreme behaviors such as excessive dieting and indiscriminate cosmetic medical interventions, posing serious risks to both mental and physical health (4, 5). Therefore, systematically elucidating the mechanisms underlying appearance anxiety and developing effective intervention strategies has become a critical task for contemporary research on university students’ mental health.

Previous studies have suggested that appearance anxiety among university students does not arise solely from dissatisfaction with one’s appearance, but is closely related to daily lifestyle patterns and the broader sociocultural environment. Unhealthy lifestyle habits may have a reciprocal relationship with appearance anxiety (6). According to self-discrepancy theory, when individuals perceive a marked gap between their ideal body image and actual body image, they are more likely to experience negative emotions such as anxiety, shame, and dissatisfaction (7). Among university students, lifestyle behaviors such as dietary control, physical activity participation, weight-loss pursuits, and appearance-related consumption may shape how individuals evaluate their bodies and appearance. Prior research has also found that irrational appearance-management behaviors, such as excessive dieting and weight-loss-related consumption, may exacerbate appearance anxiety by distorting aesthetic perceptions, intensifying body dissatisfaction, and amplifying social comparison pressure (8). Collectively, these findings suggest that appearance anxiety is closely associated with university students’ lifestyle patterns.

However, existing studies have primarily focused on offline lifestyle behaviors, such as diet, physical activity, and consumption (9–11). In the digital era, university students’ lifestyles have increasingly extended into online spaces (12). Internet use is no longer merely a tool for information acquisition or social communication, but has become an integral part of students’ daily routines, social interaction, entertainment consumption, and self-presentation (13). Against this background, Internet addiction can be regarded as a maladaptive form of online lifestyle, and its association with appearance anxiety may differ from that of traditional offline lifestyle behaviors (14). Compared with general lifestyle habits, Internet addiction may expose individuals more frequently and continuously to idealized appearances, edited body images, and algorithmically recommended appearance-related content on social media, thereby increasing the likelihood of upward social comparison and perceived body-image self-discrepancy (15, 16). From the perspective of Internet addiction theory, excessive Internet use not only reflects behavioral dependence on online environments, but may also be accompanied by attentional bias, difficulties in emotion regulation, and weakened cognitive control, making it more difficult for individuals to disengage from negative comparisons and appearance-related evaluative pressure (17). Therefore, examining the association between Internet addiction and appearance anxiety from the perspective of online lifestyle may help extend the explanatory scope of existing lifestyle research and further clarify the specific psychological processes underlying appearance anxiety among university students in the digital context (18).

Existing studies have also provided preliminary evidence for the association between Internet use and appearance-related psychological distress. For example, social media addiction may contribute to body dissatisfaction among young women through awareness of appearance-related pressure and internalization of body ideals (19). Internet addiction has also been found to be significantly associated with social appearance anxiety (20), and social comparison may play a role in the association between Internet use and appearance-related anxiety (21). In addition, previous research has shown that upward social comparison on social media is significantly associated with appearance anxiety, with self-objectification serving as a mediator and self-compassion acting as a buffering factor (21). Taken together, these findings suggest that the relationship between Internet addiction and appearance anxiety may not be merely a direct association, but should be further understood through psychological processes such as social comparison.

At the intervention level, previous approaches targeting problematic Internet use among university students have often relied on externally controlled or restrictive strategies, such as regulating daily schedules, increasing the cost of Internet use, and providing educational campaigns. However, the overall effectiveness of these approaches remains limited (22, 23). In recent years, increasing attention has been paid to the idea that, rather than merely restricting Internet use, fostering a healthy and active lifestyle and promoting the internalization of positive behaviors may be more beneficial for improving university students’ mental health (24, 25). Among these lifestyle factors, physical activity has been shown to have positive implications for body image and psychological adjustment among university students (26). According to self-determination theory (27), behavioral motivation can be conceptualized along a continuum ranging from controlled to autonomous regulation. Behaviors that are driven by personal interest, value endorsement, and self-directed choice are considered more autonomous and are more likely to promote sustained engagement and positive psychological outcomes (28). Therefore, in the context of physical activity, autonomous participation in physical activity (APPA) does not simply refer to whether individuals engage in physical activity, but rather emphasizes the extent to which such participation is based on volition, interest, personal endorsement, and intrinsic needs (29).

Based on this rationale, the present study conceptualizes Internet addiction as an important manifestation of maladaptive online lifestyle patterns among university students and examines its association with appearance anxiety (30, 31). It further investigates whether upward social comparison can explain this association. In addition, given that APPA may contribute to self-regulation, body-related perceptions, and psychological adjustment, this study further examines its moderating role in the pathway linking Internet addiction, upward social comparison, and appearance anxiety (32). Through this approach, the present study aims to provide evidence for understanding the psychological processes underlying appearance anxiety among university students in the context of digitalized lifestyles, and to offer practical implications for university-based mental health education and physical activity promotion interventions (28).

## 2 Literature review

### 2.1 Internet addiction and appearance anxiety among university students

Internet addiction refers to an uncontrolled dependence on Internet use, typically characterized by excessive online engagement, difficulty reducing use, negative emotions such as anxiety and irritability when disconnected from the Internet, and a strong dependence on virtual environments (33). University students may be particularly vulnerable to excessive Internet use because of their relatively flexible free time and still-developing self-regulatory capacity (34). According to social cognitive theory, individuals’ cognition, emotions, and behaviors are shaped through continuous interactions with environmental cues, observational learning, and self-regulatory processes (35). In the context of Internet addiction, university students may be exposed more frequently and for longer periods to carefully curated and idealized appearance-related content on social media. Such environmental cues may influence their evaluations of their own appearance through observational learning and the internalization of aesthetic standards (36, 37).

On the one hand, excessive Internet use may occupy time and energy that could otherwise be devoted to regular routines, physical activity, and daily self-management, and it may also be associated with bedtime procrastination and disrupted daily rhythms (38–40). These factors may, in turn, influence students’ perceptions of their own appearance-related condition. On the other hand, highly idealized appearance presentations on online platforms may heighten students’ attention to virtual aesthetic standards. When individuals compare their own appearance with these idealized standards and perceive a discrepancy between their actual self and ideal appearance, they may experience negative self-evaluation and appearance-related concerns (41). Compared with general Internet use, the uncontrolled and persistent nature of Internet addiction may make it more difficult for individuals to reduce exposure to such content, thereby increasing their vulnerability to appearance anxiety.

Taken together, Internet addiction may be associated with higher levels of appearance anxiety by increasing exposure to idealized appearance-related content, strengthening appearance-related cognitive evaluations, and interfering with university students’ daily self-management. Accordingly, this study proposes the following hypothesis:

*H1*: Internet addiction is positively associated with appearance anxiety among university students.

### 2.2 The mediating role of upward social comparison

According to social comparison theory, individuals have an inherent motivation to evaluate their own abilities and attributes. When objective standards are unavailable, they tend to assess themselves by comparing themselves with others (42). Upward social comparison refers specifically to the psychological process through which individuals compare themselves with others who are perceived to be superior or more aligned with socially valued standards (43). In the domain of appearance-related cognition among university students, the objects of upward comparison have shifted with the increasing digitalization of daily life. Such comparisons are no longer limited to real-life peers, but are increasingly directed toward idealized appearance images on social media that are filtered, carefully curated, and selectively presented (44).

Internet addiction, as a pattern of Internet use characterized by impaired control and persistence, may increase the frequency and intensity of university students’ exposure to idealized appearance-related content. Prior studies have shown that excessive Internet use or Internet addiction is closely associated with negative social comparison and upward social comparison tendencies (45). In social media environments, university students may frequently encounter selfies, body displays, beauty-related posts, and idealized lifestyle content shared by others, even without actively searching for appearance-related information (46). Such content may serve as salient appearance comparison targets, directing individuals’ attention to the discrepancy between their own appearance and idealized standards.

There is also a solid theoretical and empirical basis for the association between upward social comparison and appearance anxiety. When university students compare their own appearance with more idealized referents, they may perceive a discrepancy between their actual appearance and ideal appearance, which may further contribute to negative self-evaluation, body dissatisfaction, and appearance-related concerns (21). This process may be particularly relevant for university students, whose self-identity is still developing and whose self-concept may be strongly influenced by external evaluation and peer feedback (34). Therefore, frequent appearance-related upward social comparison may make individuals more attentive to perceived appearance shortcomings and may be associated with higher levels of appearance anxiety.

Taken together, Internet addiction may be associated with higher levels of appearance anxiety by increasing university students’ exposure to idealized appearance-related content and their likelihood of engaging in appearance-related upward social comparison. Upward social comparison may therefore represent an important psychological pathway linking Internet addiction to appearance anxiety. Accordingly, this study proposes the following hypothesis:

*H2*: Upward social comparison mediates the association between Internet addiction and appearance anxiety among university students.

### 2.3 The moderating role of autonomous participation in physical activity

Autonomous participation in physical activity is grounded in self-determination theory, which posits that individual behavioral motivation is shaped by three basic psychological needs: autonomy, competence, and relatedness. Among these, autonomy emphasizes whether a behavior is driven by one’s own volition, interest, and value endorsement, rather than by external pressure or coercion (27, 47). In the context of physical activity, autonomous participation refers to individuals’ ability to choose and sustain physical activity based on personal interest and internal endorsement. Previous studies have shown that higher levels of exercise autonomy are associated with greater exercise adherence and may promote body acceptance and self-identity through positive bodily experiences (48). Therefore, compared with physical activity participation per se, APPA may better capture the psychological significance of physical activity for individual adjustment.

Autonomous participation in physical activity may play a buffering role in the association between Internet addiction and upward social comparison. Internet addiction is often accompanied by more frequent and prolonged Internet use, which may increase individuals’ exposure to idealized appearance-related content on social media (49). For university students with higher levels of APPA, their engagement in physical activity is more likely to be based on self-directed choice and intrinsic interest. This may help enhance behavioral control and self-regulatory capacity, thereby reducing excessive dependence on virtual environments (50). In addition, sustained autonomous physical activity experiences may strengthen individuals’ attention to bodily function, health status, and the realistic value of the body, which may reduce their sensitivity to idealized appearance standards online. Accordingly, this study proposes the following hypothesis:

*H3a*: APPA moderates the association between Internet addiction and upward social comparison.

Furthermore, APPA may also moderate the association between upward social comparison and appearance anxiety (51). Upward social comparison may lead individuals to perceive a discrepancy between their actual appearance and idealized standards, thereby contributing to appearance-related concerns and negative self-evaluation (52). However, higher levels of APPA may help individuals develop a more stable body-related self-concept through positive bodily experiences, identification with bodily function, and emotion regulation (53). Previous studies have shown that autonomous physical activity participation is closely associated with positive body image, body satisfaction, and mental health (26, 54). Therefore, when university students engage in upward social comparison, higher levels of APPA may weaken its positive association with appearance anxiety. Accordingly, this study proposes the following hypothesis:

*H3b*: APPA moderates the association between upward social comparison and appearance anxiety.

**Figure 1:**
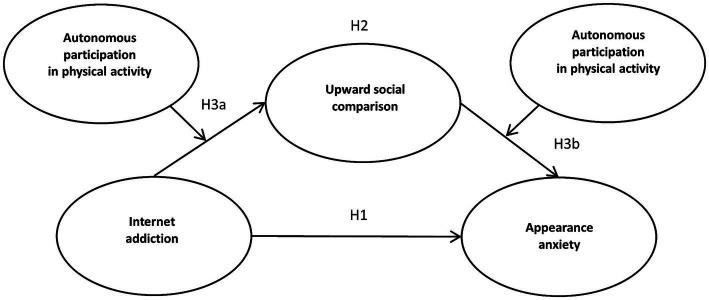
Hypothesized model of the effects of internet addiction on appearance anxiety among university students.

Taken together, although previous studies have suggested that internet addiction may influence appearance anxiety among university students, the underlying mechanisms linking internet addiction to appearance anxiety remain insufficiently clarified. Social comparison, as an important psychological basis of appearance anxiety, may be particularly salient in online contexts, where curated and idealized self-presentations can create a virtual veil that encourages comparisons extending beyond objective reality, thereby contributing to the development of appearance anxiety. However, empirical evidence regarding this process remains limited. Therefore, the present study first examines whether upward social comparison mediates the association between internet addiction and appearance anxiety among university students. Furthermore, it investigates whether this indirect pathway varies across students with different levels of APPA. By doing so, this study aims to clarify the psychological mechanism through which internet addiction contributes to appearance anxiety and to provide evidence for promoting voluntary physical activity participation among university students, reducing the negative effects of internet addiction, fostering healthier values toward appearance, and strengthening the educational role of physical activity in higher education. The hypothesized research model is shown in Figure 1.

## 3 Methods

### 3.1 Research subjects and research procedure

Shanghai-often characterized as an international fashion hub and a mega, economically developed metropolis-features a highly digitalized urban environment, which constitutes a defining aspect of its development. The city also hosts large numbers of well-known universities and attracts students from diverse backgrounds. University students in Shanghai exhibit high rates of internet use and represent a primary population that receives information through online channels, making them particularly suitable for investigating the linkage between internet addiction and appearance anxiety (55, 56). Accordingly, the present study recruited undergraduate students from five universities in Shanghai-Shanghai University, East China Normal University, University of Shanghai for Science and Technology, Shanghai Normal University, and Shanghai University of International Business and Economics—covering different institutional types. This sampling strategy included major disciplinary categories (e.g., comprehensive programs, science and engineering, humanities and social sciences, and business/management), which helps enhance the representativeness of the findings and their potential generalizability. A total of 47 questionnaire items across four established scales were administered in the survey. Following common practice in questionnaire-based research, an initial target sample size was estimated based on the item-to-respondent ratio, with 5–10 participants per item generally recommended (57). Using the more conservative criterion of 10 participants per item, the minimum recommended sample size was 470. To provide a more rigorous justification for sample adequacy, an *a priori* power analysis was also conducted using G*Power 3.1. Given that the main analyses involved multiple regression and PROCESS models, the power analysis was performed using F tests: linear multiple regression, fixed model, *R*^2^ deviation from zero. Assuming a small-to-medium effect size of *f*^2^ = 0.05, an *α* level of 0.05, a desired statistical power of 0.90, and a maximum of 10 predictors, the minimum required sample size was estimated to be 420. Data were collected via an online survey administered on Wenjuanxing (Questionnaire Star). Before completing the survey, all participants read and electronically signed an online informed consent form. They were fully informed of the study purpose, anonymity, and voluntary participation, and were explicitly advised that they could withdraw unconditionally at any stage of the study. Based on the average completion time observed in the pilot survey, questionnaires completed in less than 120 s or those with the same response selected for five consecutive items were regarded as invalid (58). After data screening, 522 valid questionnaires were retained, yielding an effective response rate of 93.7%. The final valid sample exceeded both the recommended item-to-respondent criterion and the minimum sample size required by the power analysis. This suggests that the study had adequate statistical power to test the proposed mediation and moderation models. The sample included students across all undergraduate year levels and covered a wide range of majors (e.g., science and engineering; humanities, philosophy, law; economics and management; arts and education). The sample comprised 265 men and 257 women, indicating a generally balanced gender distribution. Regarding academic standing, the sample was relatively evenly distributed across the four undergraduate year levels: freshmen (*n* = 121, 23.2%), sophomores (*n* = 162, 31.0%), juniors (*n* = 144, 27.6%), and seniors (*n* = 95, 18.2%). In terms of age, 283 participants (54.2%) were aged 18–20 years, 143 (27.4%) were aged 20–22 years, and 96 (18.4%) were older than 22 years. With respect to academic majors, science and engineering accounted for 148 participants (28.4%), humanities, philosophy, law, and related fields for 106 (20.3%), economics and management for 106 (20.3%), medicine for 69 (13.2%), arts and education for 60 (11.5%), and other disciplines for 33 (6.3%). Regarding daily internet use, 39 participants (7.5%) reported ≤3 h, 151 (28.9%) reported 3–5 h, and 332 (63.6%) reported more than 5 h per day (Table 1). This study was approved by the Ethics Committee of Shanghai University (ECSHU 2025–019) in accordance with the Declaration of Helsinki.

**Table 1:** Descriptive statistics(N = 522).

Variable	Classification	N	Percentage(%)
Gender	Male	265	50.8
	Female	257	49.2
Academic year	Freshmen	121	23.2
	Sophomores	162	31.0
	Juniors	144	27.6
	Seniors	95	18.2
Age	18 ≤ age<20	283	54.2
	20 ≤ age<22	143	27.4
	>22	96	18.4
Major category	Science and engineering	148	28.4
	Humanities, philosophy, law, and related fields	106	20.3
	Economics and management	106	20.3
	Medicine	69	13.2
	Arts and education	60	11.5
	Others	33	6.3
Daily internet use (Hour)	≤3	39	7.5
	3–5	151	28.9
	≥5	332	63.6

### 3.2 Research tools

#### 3.2.1 Internet addiction scale

In the present study, internet addiction was assessed using the Chinese-context revised version of the Internet Addiction Scale developed by Bai and Fan (59), which was adapted from Young’s (33) instrument (33). To ensure relevance to contemporary patterns of internet use among university students across diverse online contexts, the items were screened and refined. The final scale retained 15 representative items (e.g., “Based on my observation, some of my classmates feel that life is no fun without the Internet,” and “Compared with the past, I can sense that the amount of time my classmates spend online has been continuously increasing”). Items were rated on a 5-point Likert scale (1 = strongly disagree, 5 = strongly agree). A total score was computed by summing responses across all items, with higher scores indicating a greater tendency toward internet addiction. The observationally reformulated scale showed good reliability and structural validity in the present sample. The Cronbach’s *α* coefficient was 0.960, and the KMO value was 0.982, indicating good internal consistency and suitability for factor analysis. Confirmatory factor analysis (CFA) results showed good model fit, with χ^2^/df = 1.190, NFI = 0.982, RFI = 0.979, and RMSEA = 0.019. These results indicate that the reformulated items were well aligned with the hypothesized factor structure. All standardized factor loadings exceeded 0.70, suggesting that the items adequately represented their corresponding latent constructs. Taken together, these results provide empirical support for the reliability and structural validity of the observationally reformulated scale in the present sample.

#### 3.2.2 Appearance anxiety scale

Appearance anxiety was measured using the Social Appearance Anxiety Scale developed by Hart et al. (60). The scale was translated and culturally adapted to the Chinese social networking context by Chinese scholars (61) and has been widely applied in Chinese university student samples (62). To enhance applicability to contemporary social interaction contexts among university students, items were screened and refined. The final version included 14 items (e.g., “Based on my observation, some of my classmates feel nervous when taking photos,” and “Based on my observation, some of my classmates feel uneasy when they are being noticed by others”). Items were rated on a 5-point Likert scale (1 = strongly disagree, 5 = strongly agree). A total score was computed by summing responses across all items, with higher scores indicating higher levels of appearance anxiety. The observationally reformulated scale showed good reliability and structural validity in the present sample. The Cronbach’s *α* coefficient was 0.889, and the KMO value was 0.980, indicating good internal consistency and suitability for factor analysis. CFA results showed good model fit, with χ^2^/df = 1.088, NFI = 0.986, RFI = 0.983, and RMSEA = 0.013. All standardized factor loadings exceeded 0.70, suggesting that the items adequately represented their corresponding latent constructs.

#### 3.2.3 Upward social comparison scale

Upward social comparison was assessed using the Upward Comparison Subscale originally developed by Gibbons and Buunk (63) and revised by Bai et al. (64). The scale consists of 6 items (e.g., “Based on my observation, some of my classmates often compare themselves with people who are more attractive in appearance,” and “Based on my observation, some of my classmates tend to pay attention to people who look better than they do in order to gauge their own level of appearance”). Items were rated on a 5-point Likert scale (1 = strongly disagree, 5 = strongly agree). A total score was calculated by summing item responses, with higher scores indicating a stronger tendency toward upward social comparison. The observationally reformulated scale showed good reliability and structural validity in the present sample. The Cronbach’s *α* coefficient was 0.954, and the KMO value was 0.914, indicating good internal consistency and suitability for factor analysis. CFA results showed good model fit, with χ^2^/df = 1.534, NFI = 0.963, RFI = 0.961, and RMSEA = 0.032. All standardized factor loadings exceeded 0.70, suggesting that the items adequately represented their corresponding latent constructs.

#### 3.2.4 Autonomous participation in physical activity scale

APPA was measured using a revised scale primarily based on the Behavioral Regulation in Exercise Questionnaire-2 developed by Markland et al. (65), which is grounded in self-determination theory. Previous Chinese research has supported the applicability of its theoretical basis for assessing autonomy in physical activity (66, 67). In addition, items related to “autonomous choice” and “goal planning” were integrated from the Sport Motivation Scale (68), along with the “intrinsic motivation–interest/enjoyment” dimension from the Situational Motivation Scale (69). Following adaptation to the Chinese context, the final scale comprised 12 items (e.g., “Based on my observation, some of my classmates set their own exercise schedule and intensity,” and “Based on my observation, some of my classmates flexibly design exercise plans according to their physical condition”). All items were rated on a 5-point Likert scale (1 = strongly disagree, 5 = strongly agree). Total scores were computed by summing across items, with higher scores indicating higher levels of APPA. The observationally reformulated scale showed good reliability and structural validity in the present sample. The Cronbach’s α coefficient was 0.944, and the KMO value was 0.974, indicating good internal consistency and suitability for factor analysis. CFA results showed good model fit, with χ^2^/df = 1.126, NFI = 0.985, RFI = 0.982, and RMSEA = 0.016. All standardized factor loadings exceeded 0.70, suggesting that the items adequately represented their corresponding latent constructs.

### 3.3 Questionnaire adaptation procedure

Considering that some constructs examined in this study involved potentially sensitive psychological and behavioral tendencies, the original first-person self-report items were reformulated into observationally worded items. Participants were asked to evaluate the occurrence of relevant phenomena among their classmates based on their daily observations. This adaptation was intended to reduce potential self-presentation concerns and to capture students’ perceived peer-level behavioral tendencies, rather than to create new constructs or alter the theoretical meaning of the original scales.

During the adaptation process, each observational item was developed directly from its corresponding original item. The research team reviewed all items one by one according to the original dimensional definitions to ensure that the core semantic content, behavioral focus, and theoretical dimension were retained. A pilot test was then conducted to assess item clarity, comprehensibility, and contextual suitability. Minor wording revisions were made based on the pilot feedback without changing the conceptual meaning of the items.

The psychometric properties of each observationally reformulated scale, including internal consistency, KMO values, CFA model fit indices, and standardized factor loadings, are reported in the corresponding scale sections above. Overall, these results provide preliminary support for the reliability and structural validity of the adapted measures in the present sample.

### 3.4 Statistical analysis

Data were analyzed using SPSS 26.0 and PROCESS 4.1. Common method bias was first assessed using Harman’s single-factor test. Pearson correlation analyses were then conducted to examine associations among the study variables. PROCESS Model 4 was used to examine the mediating role of upward social comparison in the association between internet addiction and appearance anxiety, thereby determining whether a basic indirect relationship existed among the variables. On this basis, PROCESS Model 58 was further applied to test whether APPA moderated both the first and second stages of the mediation pathway, that is, whether the indirect effect varied across different levels of APPA (70).

## 4 Results

### 4.1 Common method bias test

Common method bias was assessed using Harman’s single-factor test. The results showed that four factors with eigenvalues greater than 1 were extracted, and the largest factor accounted for 31.220% of the total variance, which is below the recommended 40% threshold. These findings indicate that common method bias was not a serious concern in the present study.

### 4.2 Reliability test

To further assess composite reliability and convergent validity, composite reliability (CR) and average variance extracted (AVE) were calculated for each construct. As shown in Table 2, the CR values ranged from 0.890 to 0.964, exceeding the recommended threshold of 0.70. The AVE values ranged from 0.572 to 0.621, all above the recommended threshold of 0.50. These results indicate that all constructs had adequate composite reliability and convergent validity in the present sample.

**Table 2:** Composite reliability and convergent validity of the study variables (*N* = 522).

Variable	AVE	CR
Internet addiction	0.621	0.961
Upward social comparison	0.573	0.890
Appearance anxiety	0.585	0.952
Autonomous participation in physical activity	0.572	0.941

### 4.3 Descriptive statistics and correlation analysis

The correlation results (Table 3) indicated that internet addiction was positively associated with appearance anxiety among university students (*r* = 0.193, *p* < 0.01). Upward social comparison was also positively correlated with both internet addiction (*r* = 0.354, *p* < 0.01) and appearance anxiety (*r* = 0.332, *p* < 0.01). In addition, APPA was negatively correlated with internet addiction (*r* = −0.251, *p* < 0.01), upward social comparison (*r* = −0.341, *p* < 0.01), and appearance anxiety (*r* = −0.442, *p* < 0.01).

**Table 3:** Correlation analysis and the square root of AVE (*N* = 522).

Variable	M	SD	Internet addiction	Upward social comparison	Appearance anxiety	Autonomous participation in physical activity
Internet addiction	3.45	0.89	**0.788**			
Upward social comparison	3.41	0.86	0.354^**^	**0.757**		
Appearance anxiety	3.36	0.88	0.193^**^	0.332^**^	**0.765**	
Autonomous participation in physical activity	2.60	0.87	−0.251^**^	−0.341^**^	−0.442^**^	**0.756**

*N* = 522. M = mean; SD = standard deviation. **p < 0.01, bolded values indicate the square roots of the average variance extracted (AVE).

Furthermore, the square roots of the AVE values for internet addiction, upward social comparison, and appearance anxiety were each higher than their corresponding inter-construct correlation coefficients, indicating satisfactory discriminant validity and providing support for the independence and adequacy of the construct measurements.

### 4.4 Mediation analysis of upward social comparison

After controlling for gender, academic year, age, major category, and daily internet use, Model 1 indicated a significant positive association between internet addiction and appearance anxiety (*β* = 0.187, *t* = 4.389, *p* < 0.01). Similarly, Model 2 showed that internet addiction was significantly positively associated with upward social comparison (*β* = 0.341, *t* = 8.596, *p* < 0.01).

When the mediator was included, results from Model 3 showed that the direct effect of internet addiction on appearance anxiety was no longer significant (*β* = 0.082, *t* = 1.878, *p* > 0.05), whereas upward social comparison remained a significant positive predictor of appearance anxiety (*β* = 0.307, *t* = 6.780, *p* < 0.01). These findings indicate that upward social comparison fully mediates the relationship between internet addiction and appearance anxiety (Table 4).

**Table 4:** Upward social comparison as a mediator between internet addiction and appearance anxiety (*N* = 522).

Variable	Model 1 Appearance anxiety	Model 2 Upward social comparison	Model 3 Appearance anxiety
Constant	2.693**(9.597)	2.115**(8.072)	2.044**(7.156)
Gender	−0.061(−0.802)	−0.020(−0.289)	−0.054(−0.749)
Academic year	0.086(1.284)	−0.015(−0.247)	0.090(1.412)
Age	−0.070(−0.783)	0.095(1.149)	−0.099(−1.158)
Major category	0.025(1.032)	0.027(1.176)	0.017(0.723)
Daily internet use	0.010(0.158)	−0.056(−0.988)	0.027(0.459)
Internet addiction	0.187**(4.389)	0.341**(8.596)	0.082(1.878)
Upward social comparison			0.307**(6.780)
*R* ^2^	0.044	0.134	0.123
*F*	3.981, *p* = 0.000	13.285, *p* = 0.000	10.278, *p* = 0.000

**p < 0.01. The values in the brackets are *t*-values.

With the number of bootstrap samples set at 5,000 and the confidence interval at 95%, the results presented in Table 5 showed that, after controlling for gender, grade, age group, academic major, and daily Internet use duration, the total effect of Internet addiction on appearance anxiety was 0.187, with a 95% CI of [0.103, 0.271]. The 95% CI for the direct effect was [−0.003, 0.168], which included zero, indicating that the direct effect was not statistically significant. The indirect effect was 0.105, with a 95% CI of [0.069, 0.145], accounting for 56.15% of the total effect. These findings suggest that Internet addiction did not exert a significant direct effect on appearance anxiety among university students; therefore, H1 was not supported. However, upward social comparison played a significant mediating role in the association between Internet addiction and appearance anxiety, supporting H2.

**Table 5:** Upward social comparison as a mediator between internet addiction and appearance anxiety.

Pathway	Estimate	Boot LLCI	Boot ULCI	Effect proportion
Internet addiction→ Appearance anxiety (Direct effect)	0.082	−0.003	0.168	
Internet addiction→ Upward social comparison→ Appearance anxiety (Indirect effect)	0.105	0.069	0.145	56.15%
Total effect	0.187	0.103	0.271	

### 4.5 Moderated mediation analysis

A moderated mediation analysis was conducted using PROCESS 4.1 (Model 58). After controlling for gender, academic year, age group, major category, and daily internet use, the results (Table 6) indicated the following. In Model 4, internet addiction positively predicted upward social comparison (*β* = 0.274, *t* = 7.066, *p* < 0.01), whereas APPA negatively predicted upward social comparison (*β* = −0.262, *t* = −6.646, *p* < 0.01). Importantly, the interaction between internet addiction and APPA was a significant negative predictor of upward social comparison (*β* = −0.199, *t* = −4.513, *p* < 0.01). This pattern suggests that APPA negatively moderates the association between internet addiction and upward social comparison, thereby supporting H3a. In Model 5, the direct effect of internet addiction on appearance anxiety was not significant (*β* = 0.002, *t* = 0.058, *p* > 0.05). Upward social comparison significantly and positively predicted appearance anxiety (*β* = 0.195, *t* = 4.516, *p* < 0.01). In addition, the interaction between upward social comparison and APPA significantly and negatively predicted appearance anxiety (*β* = −0.187, *t* = −4.055, *p* < 0.01). These results indicate that APPA negatively moderates the relationship between upward social comparison and appearance anxiety, providing support for H3b.

**Table 6:** Tests of the moderated mediation model (*N* = 522).

Variable	Model 4 Upward social comparison	Model 5 Appearance anxiety
Constant	−0.143(−0.665)	3.302**(15.294)
Gender	−0.010(−0.149)	−0.030(−0.452)
Academic year	−0.017(−0.296)	0.079(1.344)
Age	0.090(1.155)	−0.104(−1.325)
Major category	0.030(1.387)	0.028(1.345)
Daily internet use	−0.063(−1.183)	0.025(0.476)
Internet addiction	0.274**(7.066)	0.002(0.058)
Autonomous participation in physical activity	−0.262**(−6.646)	−0.373**(−9.085)
Internet addiction × Autonomous participation in physical activity	−0.199**(−4.513)	
Upward social comparison		0.195**(4.516)
Upward social comparison × Autonomous participation in physical activity		−0.187**(−4.055)
*R* ^2^	0.231	0.262
*F*	19.269, *p* = 0.000	20.93, *p* = 0.000

**p < 0.01. The values in the brackets are *t*-values.

**Figure 2:**
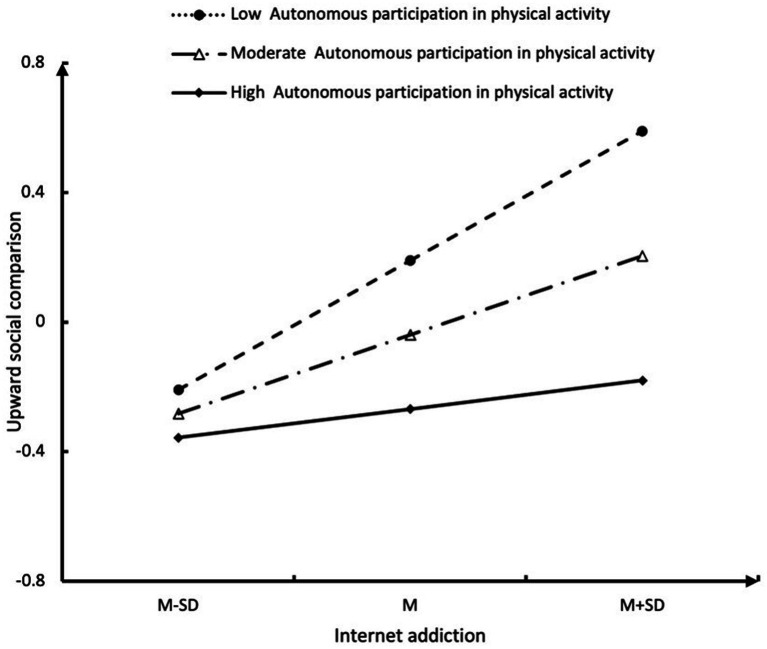
Moderating effect of autonomous participation in physical activity on the relationship between internet addiction and upward social comparison.

**Figure 3:**
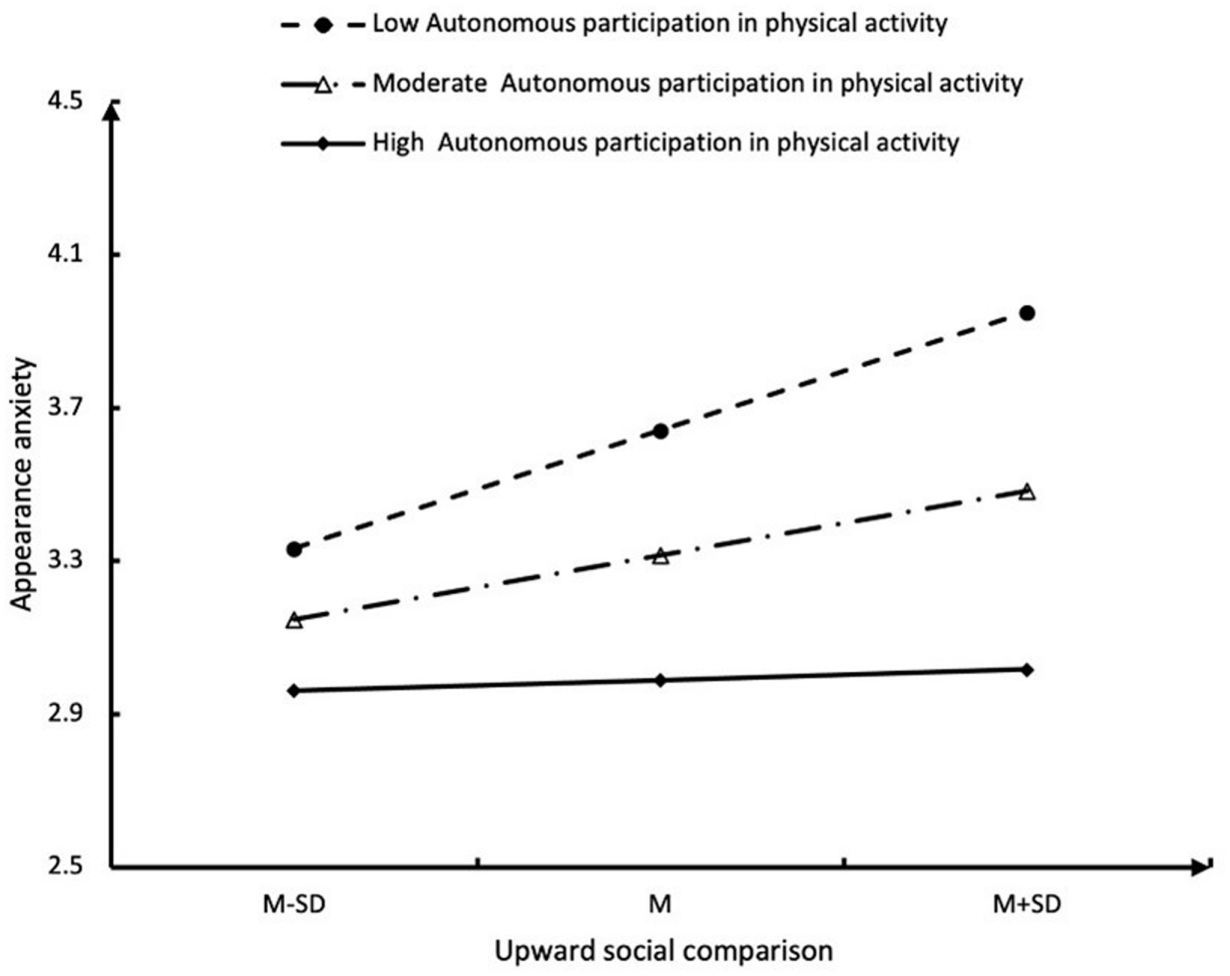
Moderating effect of autonomous participation in physical activity on the relationship between upward social comparison and appearance anxiety.

Simple slope analyses (Figures 2, 3) showed that APPA moderated both pathways. Specifically, Internet addiction was positively associated with upward social comparison, and upward social comparison was positively associated with appearance anxiety at low and moderate levels of APPA. However, both associations weakened as APPA increased and became non-significant at high levels of APPA.

## 5 Discussion

### 5.1 Internet addiction and appearance anxiety

The findings of the present study showed that, at the total-effect level, Internet addiction was significantly associated with appearance anxiety among university students. However, after upward social comparison was included in the model, the direct path between Internet addiction and appearance anxiety was not statistically significant. Therefore, H1 was not supported. It should be noted that this finding does not indicate that Internet addiction is entirely unrelated to appearance anxiety. Rather, it suggests that, within the present model, the association between the two variables may be mainly reflected through other psychological processes.

This finding differs somewhat from previous studies reporting a direct association between Internet addiction and appearance anxiety (20, 36, 37). Such inconsistency may be related to differences in model specification, sample characteristics, measurement tools, and whether mediating variables were included. Previous studies have often examined the relationship between Internet use or Internet dependence and appearance anxiety directly (71). In contrast, the present study further incorporated upward social comparison as a psychological processing variable. The results showed that, once upward social comparison was included in the model, the direct path between Internet addiction and appearance anxiety was no longer significant. This suggests that the association between Internet addiction and appearance anxiety may not primarily operate through a direct pathway, but may instead be reflected through more specific psychological processes such as social comparison.

From the perspective of university students’ Internet use, Internet addiction often involves more frequent, prolonged, and less controllable online engagement. Social media, short-video platforms, and image-based content frequently present idealized appearances, edited images, and carefully curated lifestyles, which may increase opportunities for upward social comparison (72). According to body image self-discrepancy theory, a greater perceived gap between one’s ideal body image and actual body image is more likely to be associated with negative emotional experiences (73). Therefore, rather than being directly associated with appearance anxiety, excessive Internet use may provide more contextual cues for appearance-related comparison, while upward social comparison may further relate to negative evaluations of one’s own appearance.

In addition, previous research has suggested that the relationship between Internet addiction and appearance anxiety may be bidirectional. Individuals with higher levels of appearance anxiety may also increase their Internet use to seek social approval, avoid real-life stress, or engage in appearance management (41). The present study does not deny this possibility. However, given its cross-sectional design, it cannot make firm conclusions about causal direction. Therefore, Internet addiction is more cautiously conceptualized in this study as a manifestation of maladaptive digital lifestyle patterns among university students, and the focus is placed on its association with appearance anxiety rather than on claiming that Internet addiction directly causes appearance anxiety.

### 5.2 The mediating role of upward social comparison

The present study further found that upward social comparison played a significant mediating role in the association between Internet addiction and appearance anxiety. Specifically, after upward social comparison was included in the model, the confidence interval for the direct effect between Internet addiction and appearance anxiety was [−0.003, 0.168], which included zero, indicating that the direct effect was not statistically significant. In contrast, the confidence interval for the indirect effect through upward social comparison was [0.069, 0.145], which did not include zero, and the indirect effect accounted for 56.15% of the total effect. These results suggest that, in the present model, the association between Internet addiction and appearance anxiety was mainly reflected through the indirect pathway of upward social comparison.

This finding is broadly consistent with previous research. Prior studies have shown that Internet addiction is associated with social comparison tendencies (72, 74, 75), and that social comparison, particularly upward social comparison, is closely related to body dissatisfaction and appearance anxiety (76, 77). Building on these two lines of research, the present study suggests that upward social comparison may be an important psychological pathway for understanding the relationship between Internet addiction and appearance anxiety.

From a theoretical perspective, social comparison theory provides a useful framework for interpreting this finding (42). When university students are exposed to idealized appearance presentations and curated lifestyles in online environments, they may be more likely to compare their own appearance with higher or more idealized standards (52). Frequent engagement in such comparisons may direct greater attention to the perceived discrepancy between one’s actual appearance and ideal appearance, which may be associated with higher levels of appearance anxiety (78). Social cognitive theory also suggests that external environmental cues do not automatically shape emotions; rather, their effects are often processed through cognitive appraisal (79). Therefore, the present findings support an explanatory pathway of “Internet use–social comparison–appearance anxiety,” rather than a simple direct association between Internet addiction and appearance anxiety.

It should be noted that although research on algorithmic recommendation, idealized content exposure, and social media comparison has increased in recent years, the present study did not directly measure algorithmic exposure, the frequency of appearance-related content exposure, or specific platform use (80). Therefore, algorithmic environments should be regarded only as a contextual consideration rather than a mechanism directly tested in this study. More precisely, the present findings indicate that, among university students, the association between Internet addiction and appearance anxiety was mainly reflected through upward social comparison. Whether this comparison process is driven by algorithmic recommendation, platform content features, or individuals’ active content selection remains to be further examined in future research.

### 5.3 The moderating role of autonomous participation in physical activity

The results of the present study showed that APPA moderated the associations among Internet addiction, upward social comparison, and appearance anxiety. Specifically, the interaction between Internet addiction and APPA was negatively associated with upward social comparison (*β* = −0.199, *t* = −4.513, *p* < 0.01), and the interaction between upward social comparison and APPA was also negatively associated with appearance anxiety (*β* = −0.187, *t* = −4.055, *p* < 0.01). These findings indicate that higher levels of APPA were associated with weaker positive associations between Internet addiction and upward social comparison, and between upward social comparison and appearance anxiety. Thus, H3a and H3b were supported.

The simple slope analyses further showed that, at low and moderate levels of APPA, Internet addiction was positively associated with upward social comparison, and upward social comparison was positively associated with appearance anxiety. However, both associations weakened as APPA increased and became non-significant at high levels of APPA. It should be noted that this pattern should not be interpreted as indicating that the moderating effect was stronger in the low-autonomy group or absent in the high-autonomy group. Rather, the results suggest that the strength of these associations varied across different levels of APPA, with the positive associations becoming weaker as autonomous participation increased.

This finding can be interpreted in light of self-determination theory. According to this theory, behaviors that are more strongly driven by interest, value endorsement, and self-directed choice are more autonomous and are more likely to be associated with adaptive psychological outcomes (27). In the context of physical activity, autonomous participation does not merely refer to whether individuals engage in exercise, but emphasizes whether such engagement is based on volition, interest, and internal endorsement. The present results showed that, at high levels of APPA, the associations between Internet addiction and upward social comparison, as well as between upward social comparison and appearance anxiety, were not significant. This suggests that APPA may be related to lower sensitivity to negative comparison and lower vulnerability to appearance-related concerns.

Compared with previous research emphasizing the positive role of physical activity in improving body image and reducing anxiety (81, 82), the present findings further suggest that the psychological significance of physical activity may depend not only on its frequency, intensity, or type, but also on the extent to which individuals participate autonomously. Higher levels of APPA may help university students develop more positive bodily experiences and self-evaluations, making them less dependent on appearance-based standards when evaluating their self-worth (83). Accordingly, when students encounter upward comparison cues in online environments, higher APPA may be associated with a weaker comparison–anxiety link.

Nevertheless, these findings should be interpreted with caution. Because the present study used a cross-sectional design, it cannot establish that APPA directly reduces the effects of Internet addiction or upward social comparison. A more appropriate interpretation is that APPA showed a protective association in the present model: higher levels of APPA were associated with weaker Internet addiction–upward social comparison and upward social comparison–appearance anxiety pathways. Future longitudinal or intervention studies are needed to examine whether increasing APPA can reduce upward social comparison tendencies and appearance anxiety among university students in online contexts.

In summary, the findings suggest that APPA may be an important boundary condition in understanding the associations among Internet addiction, social comparison, and appearance anxiety among university students. Rather than merely emphasizing increased physical activity volume, future university-based mental health and physical activity interventions may benefit from fostering students’ APPA based on interest, value endorsement, and self-directed choice.

## 6 Conclusion

The present study yields two core conclusions. First, internet addiction does not directly lead to appearance anxiety among university students; instead, it exerts an indirect influence by intensifying upward social comparison as a key cognitive pathway. Second, APPA functions as a critical personal protective factor and demonstrates dual moderating effects on the pathway internet addiction → upward social comparison → appearance anxiety. Importantly, this buffering pattern is not uniform but shows pronounced heterogeneity and nonlinearity. Specifically, participants with low and moderate levels of APPA exhibited significant buffering effects, attenuating the extent to which internet addiction activates upward social comparison and weakening the translation from upward social comparison to appearance anxiety, with stronger buffering observed at lower levels of autonomy. In contrast, no significant moderation emerged in the high-autonomy group, likely because their psychological resources had reached a relatively saturated level, leaving limited room for additional buffering to be observed.

## 7 Limitations and educational implications

Several limitations should be acknowledged. First, the present study adopted a cross-sectional survey design, which prevents firm causal inferences among the variables and makes it difficult to capture the dynamic changes and potential bidirectional association between Internet addiction and appearance anxiety. Future research could employ longitudinal designs or cross-lagged panel models to further examine the temporal ordering and reciprocal relationship between Internet addiction and appearance anxiety. Second, although the observationally reformulated items were developed based on the original scales and were supported by content review, pilot testing, and CFA, the present study did not administer both the original self-report version and the observationally reformulated version simultaneously. Therefore, strict measurement equivalence between the two versions could not be directly tested. Future studies may compare the two versions using measurement invariance testing or adopt multi-informant designs to further examine the cross-method validity of the adapted measures. Third, although the use of observationally worded items may help reduce participants’ self-presentation concerns when responding to socially sensitive constructs, the data were still based on participants’ subjective perceptions and questionnaire responses. Therefore, the findings may still be affected by common method bias, social desirability, or individual differences in observing and interpreting peers’ behaviors. Future studies could incorporate behavioral data, interview materials, peer reports, or teacher assessments to enhance the objectivity and robustness of the findings. Forth, the participants were recruited from universities in Shanghai, which may limit the generalizability of the findings to university students in other regions or cultural contexts. Future research should include multi-region and cross-cultural samples to examine whether the proposed model is applicable across different educational, social, and cultural settings. Finally, to ensure the clarity of the core mechanism tested in this study, we did not further distinguish between different types of Internet addiction, social media platform characteristics, the degree of appearance-related content exposure, or specific attributes of physical activity. This may limit the granularity of the interpretation. Future research could use multi-center samples, mixed-method designs, and more refined measurements to further extend and validate the present findings.

The findings yield several actionable educational implications. Universities may reduce the automation of upward social comparison by incorporating media literacy education that helps students critically evaluate idealized online appearance cues. In addition, higher education institutions may optimize physical education through a tiered intervention framework, developing differentiated strategies tailored to students with varying levels of autonomy in physical activity, thereby enhancing their psychological capital for resisting appearance anxiety. Finally, practical guidance for students’ self-adjustment should emphasize replacing excessive online dependence with autonomy-driven physical activity and fostering a more diversified understanding of body value that prioritizes functionality, health, and well-being over narrow appearance-based standards.

## Data Availability

The raw data supporting the conclusions of this article will be made available by the authors, without undue reservation.
